# Compost Soil Microbial Fuel Cell to Generate Power using Urea as Fuel

**DOI:** 10.1038/s41598-020-61038-7

**Published:** 2020-03-05

**Authors:** Verjesh Kumar Magotra, Sunil Kumar, T. W. Kang, Akbar I. Inamdar, Abu Talha Aqueel, Hyunsik Im, Gajanan Ghodake, Surendra Shinde, D. P. Waghmode, H. C. Jeon

**Affiliations:** 1 Nano Information Technology Academy, Dongguk University-Seoul, Jung-Gu, 100715 Seoul, South Korea; 2 Division of Physics and Semiconductor Science, Dongguk University, Jung-Gu, 100715 Seoul, South Korea; 3 Department Biological and Environmental Science, College of Life Science and Biotechnology, Dongguk University-Seoul, Ilsandong-gu, 10326 Goyang-si, Gyeonggi-do Republic of Korea; 4 Analytical Chemistry and Material Science Research Laboratory, Department of Chemistry, Shivaji University, Kolhapur, 416004 Maharashtra India; 5 Department of Physics, Indira Gandhi University, Meerpur, 122502 Rewari, Haryana India

**Keywords:** Microbial ecology, Bioenergetics, Applied microbiology, Fuel cells, Biofuels

## Abstract

The acute problem of eutrophication increasing in the environment is due to the increase of industrial wastewater, synthetic nitrogen, urine, and urea. This pollutes groundwater, soil and creates a danger to aquatic life. Therefore, it is advantageous to use these waste materials in the form of urea as fuel to generate power using Microbial Fuel Cell (MFC). In this work, we studied the compost soil MFC(CSMFC) unlike typical MFC with urea from the compost as fuel and graphite as a functional electrode. The electrochemical techniques such as Cyclic Voltammetry, Chronoamperometry are used to characterise CSMFC. It is observed that the CSMFC in which the compost consists of urea concertation of 0.5 g/ml produces maximum power. Moreover, IV measurement is carried out using polarization curves in order to study its sustainability and scalability. Bacterial studies were also playing a significant role in power generation. The sustainability study revealed that urea is consumed in CSMFC to generate power. This study confirmed that urea has a profound effect on the power generation from the CSMFC. Our focus is to get power from the soil processes in future by using waste like urine, industrial wastewater, which contains much amount of urea.

## Introduction

The rapid increase in power consumption and various environmental issues have compelled the research community to identify new sources of renewable energy. It is pertinent to discover new renewable resources^1,4^. In this pursuit, energy storage devices such as fuel cells, which are mostly powered by organic compounds, can be useful tools. Urea Fuel cells available in the liquid state are not sustainable and portable^1^. However, in soil-based Microbial Fuel Cell (MFC) use natural bacteria or secreted enzymes to break down the fuel, typically to generate electricity from the soil. In MFCs, bacteria and enzymes to act as biocatalysts to produce electricity^1,2^. Until now the reported liquid state MFCs associated with safety concerns mainly related to toxicity, shifting, leakage, handling and degrading fastly in the liquid state. Moreover, additional precautions are needed to prevent exposure to gaseous NH_3_ due to volatilisation of the liquid fuel. Therefore, the solid-state materials like soil compost are preferred to overcome the risk, as mentioned above for stable behaviour.

Among element of urine, urea is a suitable fuel for MFCs. It is an advantage for the soil-based system to go through the natural processes by following nitrification and denitrification in the nitrogen cycle by ammonification to nitrogen (N_2_) formation in soil^3^. The soil itself is a source of many bacteria and microorganisms in aerobic and anaerobic forms^4–8^. Urea and ammonium are sources of nitrogen, and the density of urea is higher as compared to other nitrogen sources^2,3,9^. Urea when comes in contact with the soil while hydrolysis releases urease enzymes working as a catalyst with bacteria. Therefore, soil systems can be a neutral medium to transport electrons and protons easily in an eco-friendly medium for power generation and maintain the ph level for the proper working of the MFC^9–15^. Power generation from urea as fuel is shallow in the liquid state, as shown in various studies done previously^1–6^. However, there is no other technology at present, which can generate electricity from a soil-based MFC, using urea as fuel in compost. The conventional electrodes, like gold, platinum, and palladium-based catalysts, routinely used in the fuel cell industry, are expensive. In comparison, graphite electrodes are cheaper and stable; moreover, it is an excellent electron collector for MFCs. It is a purely microbial system the soil itself works as separator, mediator, and ionic conductor to promote electron and protons in the device^16–25^. It is noted that this is an entirely soil-based system, which we are analysed first-time using compost soil in MFC with urea as fuel unlikely to the typical electrochemical MFCs. The vision of this paper shown in the supplementary information, Fig. S1.

In this paper, we studied solid-state microbial fuel cell using urea as fuel from compost to generate power. Best of the author’s knowledge, this is the first report of the compost soil microbial fuel cell (CSMFC) in which urea is used as fuel. The urea concentrations studies from 0.1 g/ml to 0.5 g in the soil to justify the role of urea in CSMFC. Besides, urea electrolysis and hydrolysis confirmed its utility as an energy source from compost soil.

## Results and Discussion

**Figure 1:**
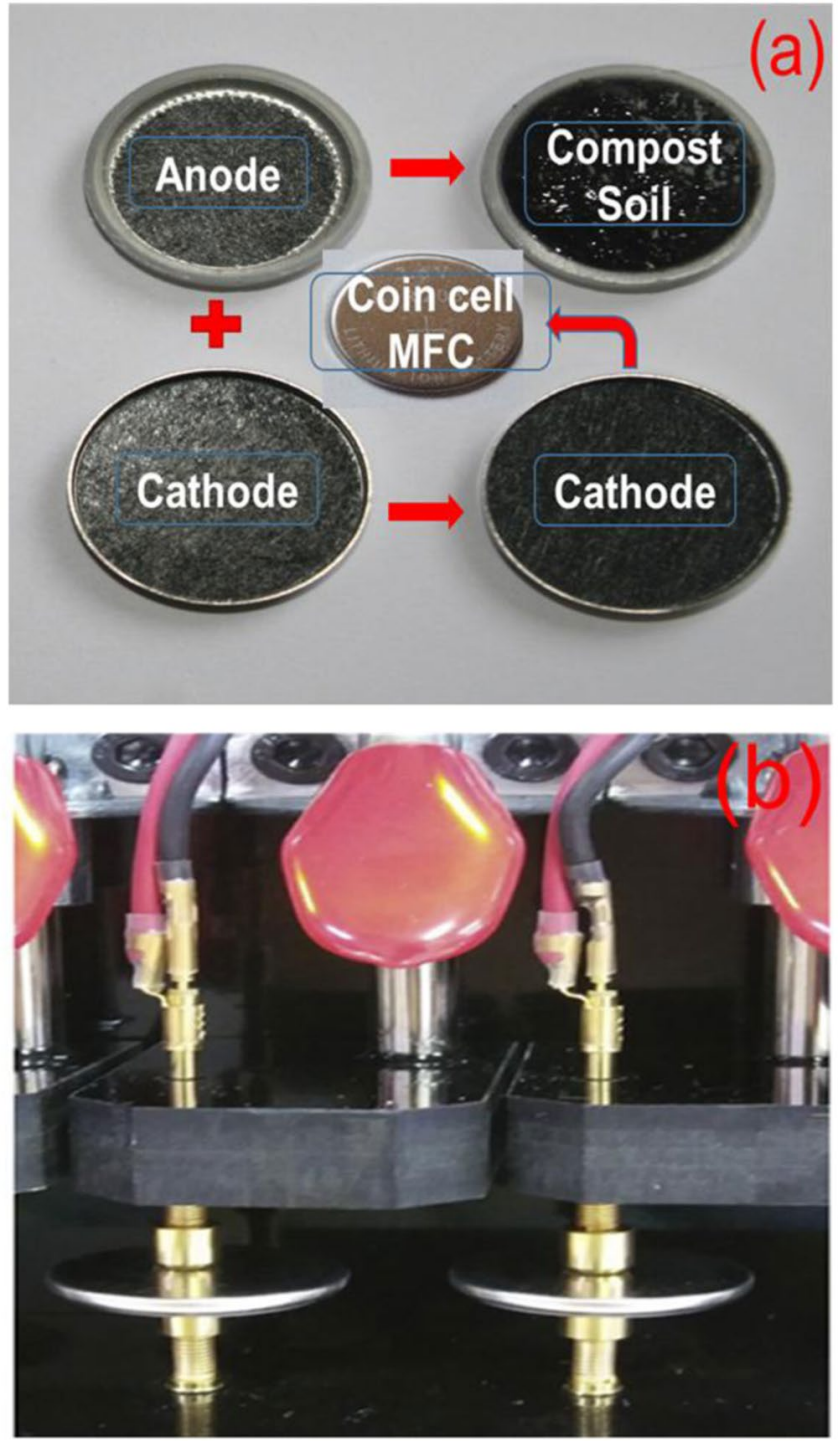
(**a**) Schematic illustration of the coin cell system used to study compost soil microbial fuel cell, (**b**) Actual photograph of the measuring system used.

Fig. 1(a) shows the schematic diagram of the cell fabricated to test the CSMFC using coin-cell type (CR 2032) system, which is normally used for battery study. The coin cell has typical dimensions such as a thickness of the cell is 0.20 cm, diameter 0.32 cm, the surface area of the coin cell is 3.14 cm^2^ ^26–28^. Graphite electrodes are used as an anode and cathode separated with compost soil. Fig. 1(b) shows a photograph of the real testing unit used in this study. The surface texture of the soil was studied using SEM measurements is shown in the supplementary information Fig. S2. The detailed experimental procedure is provided in the experimental section.

**Figure 2:**
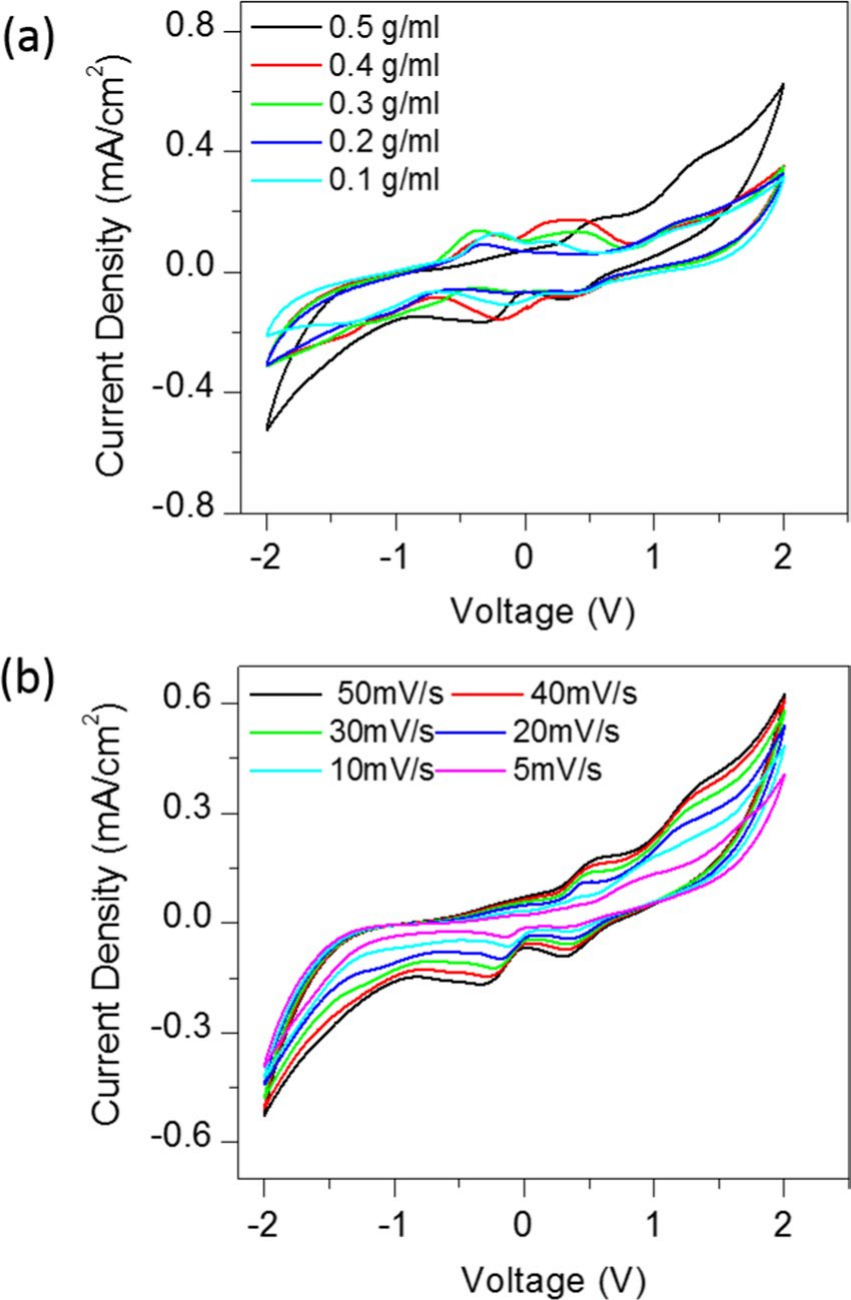
(**a**) Bipolar CV studies shows the effect of the urea fuel concentration on compost soil (**b**) CV curves with various Scan rates.

Fig. 2(a) shows the cyclic voltammograms of CSMFC with different urea concentrations, at a scan rate of 50 mV/Sec. The urea concentration in CSMFC is varied from 0.1 g/ml to 0.5 g/ml. The optimisation of the CSMFC is addressed by considering peak current and onset potentials during the oxidation and reduction process. The electrocatalytic activity was highest at a concentration of 0.5 g/ml of the urea fuel. This result indicated that urea fuel affects the compost soil directly for power. However, the redox potential peak for urea in the bipolar measurements fell in the range between 0 to ±0.6 V. It is similar to the values reported in the literature for urea, urine and near to ammonium redox potentials^4^. Urea and ammonium, ions both related to each other as sources of nitrogen^4^.as mentioned in the soil process of nitrification and denitrification of the compost^4^ Fig. 2(b) shows CV curves of the CSMFC prepared at a urea concentration of 0.5 g/ml at a different scan rates ranging from 5 mV/sec to 50 mV/sec. It revealed that the urea sample has scan-rate dependent behaviour. With an increasing scan rate, the current density also enhances. The device changed from quasi-reversible to a constant state showing positive polarity. The scan rate, showing the electrochemical reaction on the active electrode surface, occurred due to a diffusion-controlled process, according to the Randles-Sevcik model^4,22,23^. Thus, the system is suitable for both the power generation purpose and cleaning urea related waste materials. Urea as fuel in 0.5 g/ml compost-based, sample selected for further studies. The linear increase of power with respect to the scan rate and concertation of the CSMFC similarly like typical MFC.

**Figure 3:**
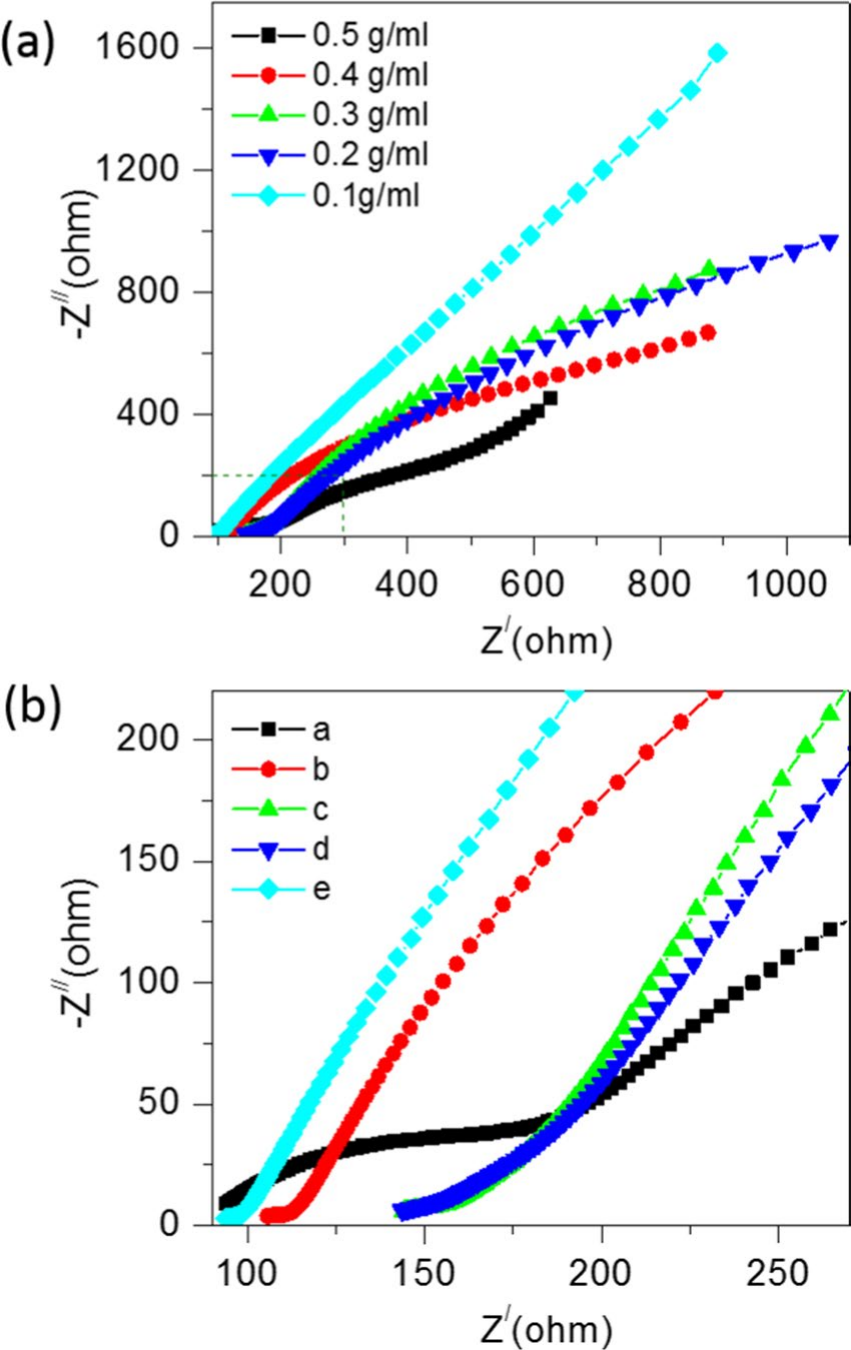
(**a**) EIS study to show the effect of the urea fuel concentrations with compost soil (**b**) Enlarged view of the EIS curves of the compost soil samples.

Electrochemical impedance spectroscopy (EIS) measurements are further performed to investigate the electrochemical behaviour of the soil compost with urea as fuel. The real and imaginary impedance was obtained for a frequency range of 0 Hz to 10,000 Hz for the applied active field. Fig. 3(a) shows Nyquist plots of CSMFC prepared at different concentrations follows a similar trend observed in the CV results. Lower impedance has higher redox potential and vice- versa. The slope of the straight line explained the mechanism in the low-frequency regions. It shows the role of Warburg impedance (W), which corresponds to the electrolyte diffusion in the urea-based material for the soil sample. The intersection of the curve from CV measurements at the real part of Z/Ohm shown in Fig. 3(b) representing the solution resistance (R_S_), the small semicircle in the enlarged view of the impedances region shows the charge transfer resistance (R_CT_) between the working electrode/electrolyte interfaces. Additionally, R_CT_ depended upon the concentration of urea. The lowest impedance was observed at a level of 0.5 g/ml. Thus, lower the urea concentration level higher was the impedance^4^.

**Figure 4:**
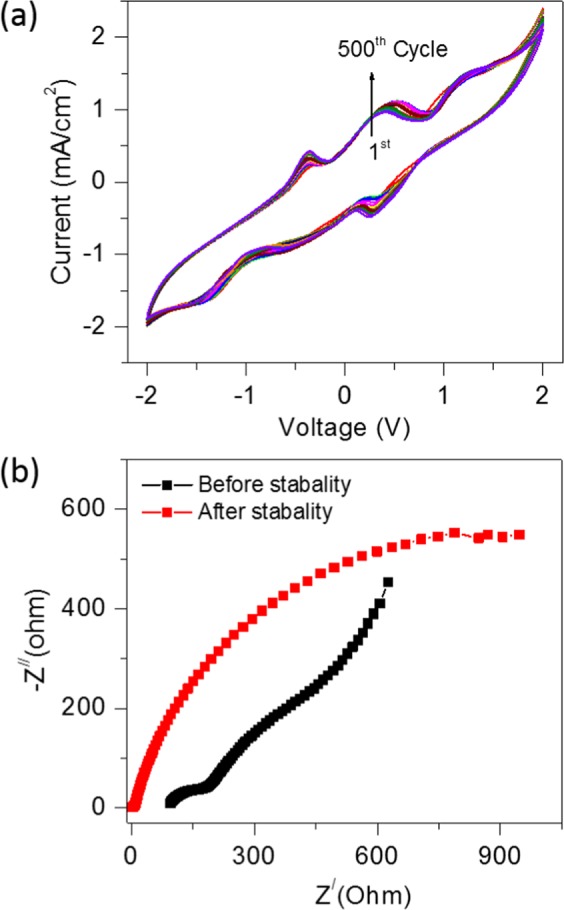
Cyclic stability of the compost soil with single shot of fuel (**a**) Cyclic stability plotted for 500 cycles (**b**) EIS studies before stability and after stability for 500 cycles.

The cyclic stability study performed for the optimisation of CSMFC prepared at a urea concentration of 0.5 g/ml. Fig. 4(a) shows the CV curves recorded for 500 cycles using a single shot of 0.5 g/ml urea fuel in compost sample. All the experiments of the stability test are performed at room temperature. The reversible behaviour of the CV curves without a change in the shape suggests the outstanding stability of the CSMFC for over 500 cycles. Fig. 4(b) shows the Nyquist plot of CSMFC showing the electrochemical behaviour before and after 500 cycles. The slope of the straight-line portion in the low-frequency region shows the Warburg impedance (W), the semicircle in the high-frequency region shows the R_CT_ at the working electrode-electrolyte interface that is caused by the faradaic-redox reaction of the electrode. After 500 cycles, R_CT_ increased significantly, which correlated with the structural degradation of the urea fuel cell. During the cycling process. The cell gradually comes at dry state after completing 500 cycles, causing an increase of impedance. Due to gradual urea fuel degradation, the power peak declines and the cell impedance increases.

**Figure 5:**
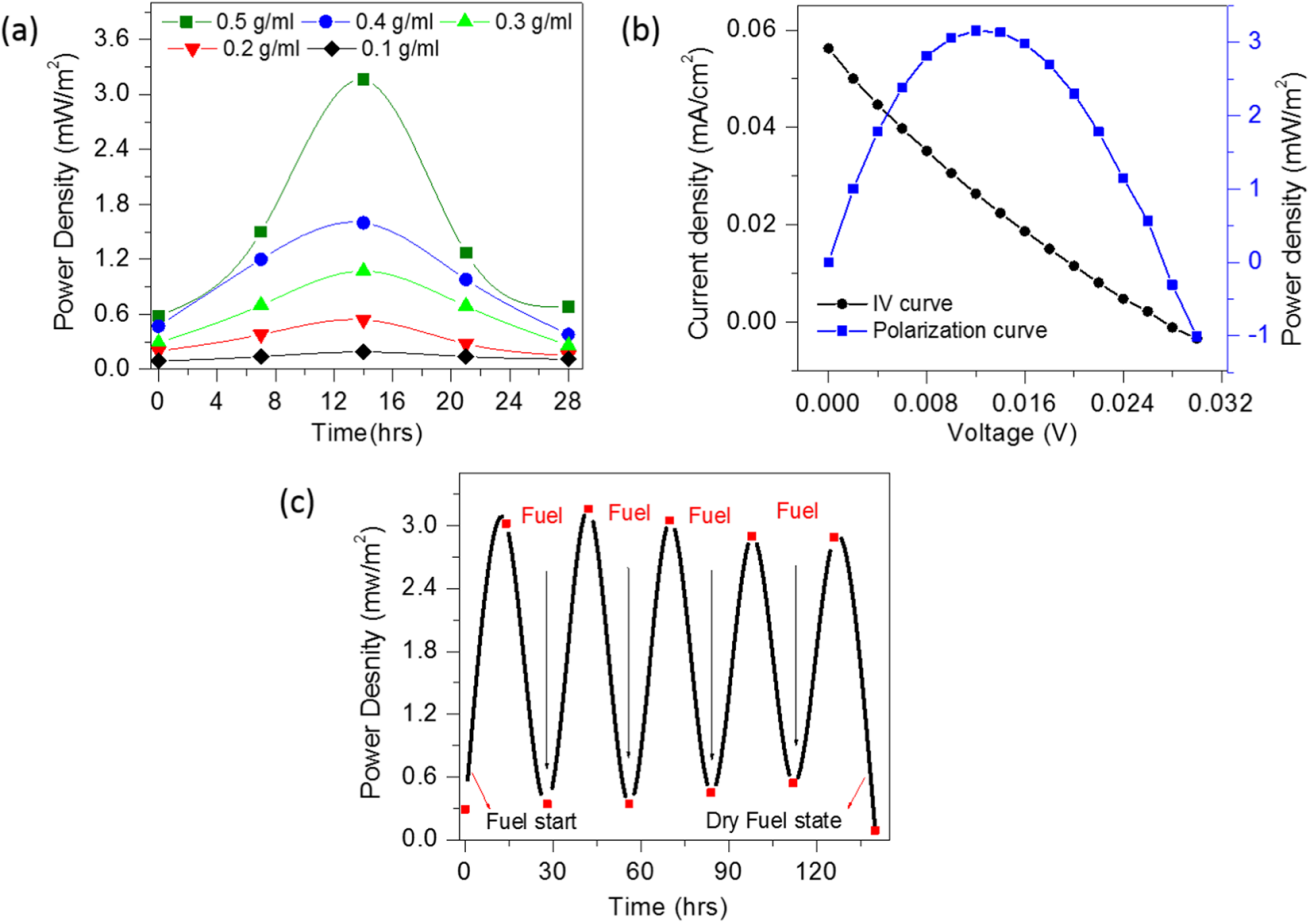
Gr/Gr Electrodes Keithley (I–V) measurement data (**a**) Urea fuel concentration variation 0.1 g/ml to 0.5 g/ml with compost soil coin cell samples (**b**) Polarization curve of the CSMFC (**c**) sustainability study of the CSMFC prepared at 0.5 g/ml urea fuel.

The I–V measurements were performed from 0 to 28 hours to study the electrocatalytic activity of the CSMFC prepared by using different urea concentrations. Fig. 5(a,b) shows a power density curve with respect to the operation time and IV curves of CSMFC. It is seen that the CSMFC activated nearly about 14 hours generating maximum power. It also noted that the power density increases with increase in the urea concentration from 0.1 to 0.5 g/ml. The maximum power density is found to be 3.16 mW/m^2^ at 0.5 g/ml of the concentration. At mass transport region (i.e. higher current) the value of voltage is low, and inactivation region(i.e. low current) the value of voltage is higher giving low power densities whereas, in the ohmic region, the power density is at its maximum value hence the power density curve symmetrical. Although the power density obtained in this study is inferior to the other systems reported in the literature, this is an entirely new system such as CSMFC unlike to the liquid-based MFCs^1–6^. On the other hand, it has some other advantages such as it is sustainable, reusable, non-toxic, cheap, eco-friendly and available easily on the Earth crust.

The CSMFC has been refuelled several times after every 28 hours and power generation was monitored to assess its stability. The results showed the stable functioning of the cell for the measured period of up to 140 hours. In order to study the consumption of urea, we also performed IV measurements in which urea was injected as fuel in regular interval of time and its power density is calculated. Please see following Fig. 5(c) of generated power density versus time. Initially, we have injected urea fuel and left for the activation. It is seen that it was activated after 14 hours after fuelling, showed maximum power, then after power decreases. After refuelling it in 2^nd^ cycle with urea, the power again increased to its maximum. This clearly indicates that the urea is consumed in CSMFC to generate power.

**Figure 6:**
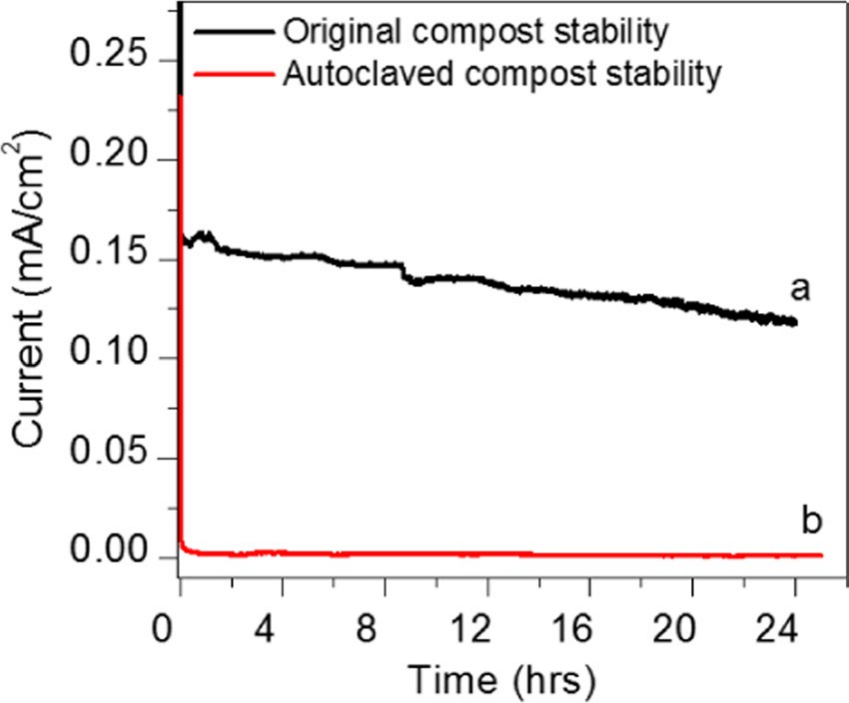
Comparsion durability test at 0.3 V between standard compost sample and autoclaved sterilization at (120 °C) treated compost sample.

The chronoamperometry, study was performed for the optimisation of the durability test for soil compost sample with a single shot of urea 0.5 g/ml fuel at 0.3 V. While constantly monitoring the sample Fig. 6 shows that the electro-oxidation activity of the compost soil sample reached a maximum current density of (0.15 mA/cm^2^). The current slowly decreased by urea oxidation. In order to know the activity of the bacteria and enzymes present in the compost, we prepared CSMFC using autoclaved compost (which kills the bacteria and enzymes). The sample was autoclaved at 120 °C killing the bacteria and enzymes from the compost. From the Fig. 6, it is seen that the current was gradually decreased to 0.012 mA at 0.3 V, as compared to the standard compost sample, which was ten times lesser than that of the soil compost. The comparison shows that the bacterial effect involved in the loss of the current in the autoclaved sample.

**Figure 7:**
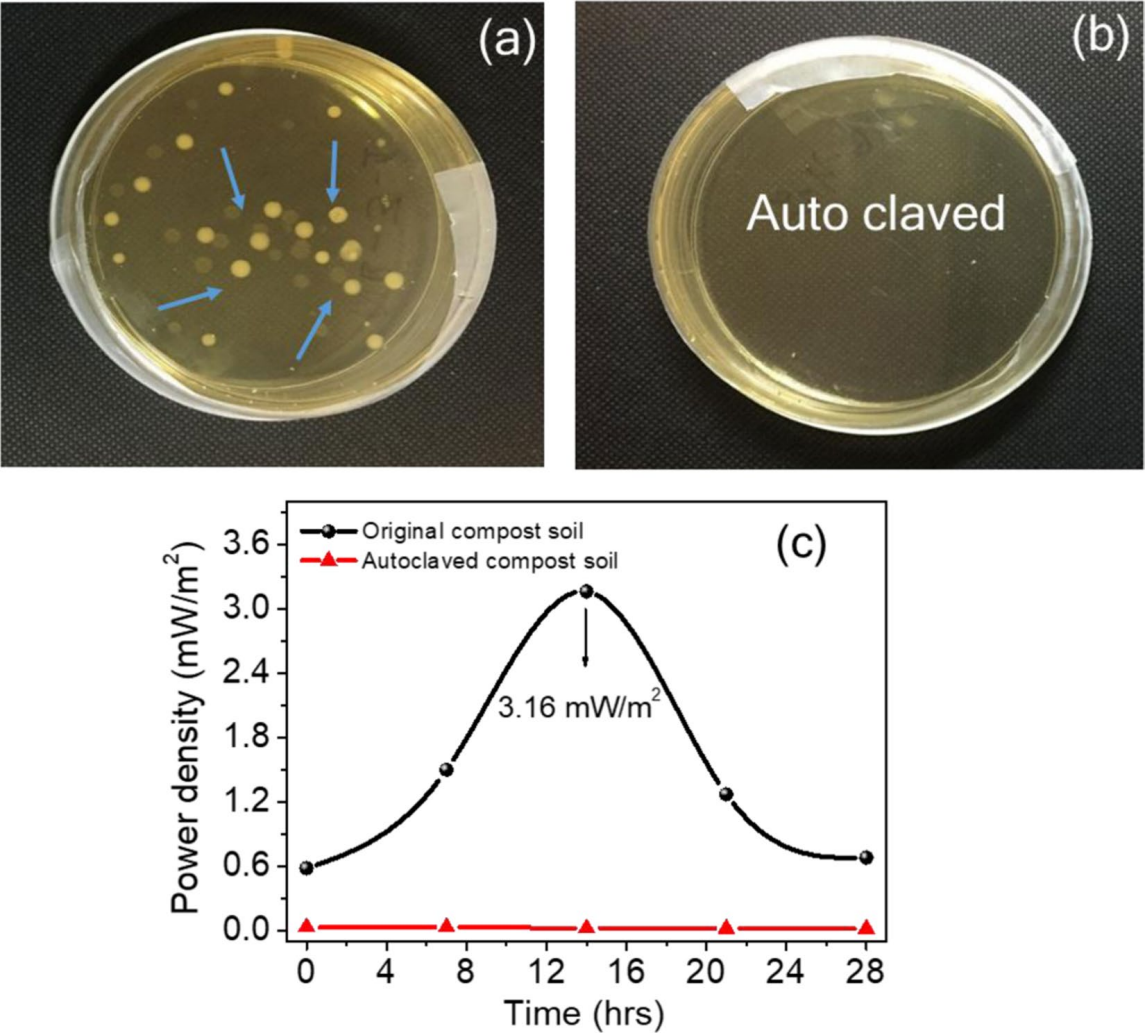
Effect of the bacterial study for compost soil coin samples (**a**) Growth of the bacterial colonies present (**b**) Growth of colonies absent (**c**)The (I–V) study showing the effect of the bacteria.

In the bacterial studies, compost soil demonstrates the role of bacteria and enzymes in the functioning of the CSMFC. The compost soil sample was sterilised by autoclave treatment, and the power generated by these cells were compared with those that were not sterilised. Fig. 7(a), while the first sample contained bacteria, from the CSMFC. Fig. 7(b) The second sample that was autoclaved at 120 °C contained no live bacteria. This was evident from Fig. 7(a),(b), which shows the bacterial growth in agar plates after 28 hours. While bacterial colonies were visible in the plates, as shown in Fig. 7(a) and no colonies were found in the autoclaved sample shown in Fig. 7(b). These results suggest the role of bacteria and enzymes in enhancing electricity generation in the CSMFC. Fig. 7(c) shows the Keithley (I–V) measurements. The CSMFC showed a maximum power density of 3.16 mW/m^2^, and the autoclave treated sample was only 0.03 mW/m^2^. Thus this study clearly indicated the role of urea in power generation, due to the effective role of soil in CSMFC^2,19^. The similarity in the results obtained from bacterial studies and (I–V) measurements suggesting that bacteria, enzymes played an essential role for the power generation. The plates were photographed, and the soil bacteria were examined for a longer time up to 84 hours (see Supplementary Information Fig. S3).

In the mechanism, the soil is known to act as an electrocatalyst^4^. Similar to bacteria and enzymes, the soil may also catalyse the oxidation of urea. Due to the addition of the nitrogen in the soil, the chemical reaction enhances the pH from 5.5 to an alkaline pH in the range of 8–9. The *V*_max_ for the high-affinity response reaction (N_2_O → NO → N_2_) showed a relatively small peak at pH 6.5, followed by first a decline and then a sharp increase in the pH to 9.5^20^. Urea is food for the bacteria; urea stimulates bacteria to release urease^19,24^. When urea was hydrolysed, it generates ammonia, ammonium ions (NH_4_^+^ ions). Compost soil performs ammonification through nitrification and denitrification process to reach to release (N_2_) as the last product while supplying protons and electrons. When urea was hydrolyzed in the soil releases the urease enzyme, it generates ammonia, ammonium ions (NH_4_^+^ ions) later. Following this, ammonification and volatilization lead to nitrification and denitrification process^2,3,19^.

Reaction 1 converts ammonia to the intermediate, hydroxylamine, and is catalyzed by the enzyme ammonia monooxygenase. Reaction 2 converts hydroxylamine to nitrite and is catalyzed by the enzymes hydroxylamine oxidoreductase^29^.

The operating mechanism of CSMFC is given below,

### Anode reaction

Urea releases the urease enzymes by hydrolysis case A and the role of urea in compost soil mechanism, as mentioned below in case B.

$${\rm{A}})\,{\rm{CO}}{({{\rm{NH}}}_{2})}_{2}+6{{\rm{OH}}}^{-}\to {{\rm{N}}}_{2}+{{\rm{CO}}}_{2}+{{\rm{5H}}}_{2}{\rm{O}}+6{{\rm{e}}}^{-}$$

$${\rm{B}})\,{\rm{CO}}{({{\rm{NH}}}_{2})}_{2}+{{\rm{H}}}_{2}{\rm{O}}\to 2{{\rm{NH}}}_{3}+{{\rm{CO}}}_{2}$$

1 $${{\rm{NH}}}_{3}+{{\rm{O}}}_{2}+2{{\rm{e}}}^{-}\to {{\rm{NH}}}_{2}{\rm{OH}}+{{\rm{H}}}_{2}{\rm{O}}$$

2 $${{\rm{NH}}}_{2}{\rm{OH}}+{{\rm{H}}}_{2}{\rm{O}}\to {{\rm{NO}}}_{2}^{-}+5{{\rm{H}}}^{+}+4{{\rm{e}}}^{-}$$

$${{\rm{NH}}}_{4}^{+}+{{\rm{NO}}}_{2}^{-}\to {{\rm{N}}}_{2}+2{{\rm{H}}}_{2}{\rm{O}}$$

### Cathode reaction

 

$${{\rm{NH}}}_{3}+{{\rm{H}}}_{2}{\rm{O}}\to {{\rm{NH}}}_{4}^{+}+{{\rm{OH}}}^{-}$$

### The overall reaction for anode and cathode

 

$$2{\rm{CO}}{({{\rm{NH}}}_{2})}_{2}+3{{\rm{O}}}_{2}\to 2{{\rm{N}}}_{2}+2{{\rm{CO}}}_{2}+4{{\rm{H}}}_{2}{\rm{O}}$$

The equations confirmed the combined mechanism for both compost soil and urea fuel enhances the power generation. Due to urea fuel in liquid state dissolved in compost soil so that bacteria and enzymes uptake, then generate electricity in the CSMFC.

Either urea or urine can be directly used as fuels to produce power in fuel cells without a membrane or with membrane^1^. Oxidation of urea to nitrogen gas, carbon dioxide results in the generation of ammonia or ammonium ions, which are converted to carbamate or carbonic acid CO (OH)_2_, as reported in the literature. In the process of ammonification, ammonium ions can be oxidized by two classes of bacteria (*Nitrobacter* and *Nitrosomonas)* to NO_3_ (nitrate) with an unstable intermediate NO_2_ (nitrite) in a process called nitrification, which eventually produces nitrogen (N_2_)^4,19,20,24^. This study confirmed that urea has a profound effect on the power generation from the CSMFC. Our focus is to get power from the compost soil process in future by using waste like urine, industrial wastewater, which contains much amount of urea. However, the real composted feedstock contains diverse components other than urea, which may slightly vary the power generation results.

## Conclusion

The multifunctional role of soil-based urea microbial fuel cell systems acts as a mediator, source of electronic bacteria, and supply’s nutrient & water to the microbes was demonstrated. This CSMFC is shown to generate power from using urea as fuel. As a MFC, it does not have the disadvantages typically associated with liquid-state MFCs. Moreover, it can be a cost-effective alternative. Because any compost, which is cheap and abundant, it is a viable candidate for the MFC. The results of the cyclic voltammetry (CV) and (IV) measurements reinforce each other and indicate that soil microbes with addition to urea, as fuel in the soil is the source of charges in the MFCs. A concentration of 0.5 g/ml concentration of urea fuel in the soil was found to be optimal, producing a power density of 3.16 mW/m^2^. Exploiting the different types of energy-generating soil bacteria and enzymes already present in the soil, our novel design for the coin cell for generating energy and in future side by side checking with single chamber CSMFCs using compost-based urea as a fuel cell is suitable for sustainable power generation. Moreover, in the process of power generation, it can also remedy the different waste materials, urine, urea, industrial wastewater, at the big scale it may work in for the cleaning purpose. This study demonstrates advancement in the field of CSMFC technology, by providing an eco-friendly, sustainable, and cheap energy generation technology with plenty of scope of research in the future directed towards compost soil-based organic MFCs.

## Methods

### Sample preparation

For this study, the compost soil supplied by Seoul Seung Jin Fertilisers Pvt Ltd., Korea is used. The coin cells of type CR2032 are used to fabricate CSMFCs similarly like it was previously reported in our report^7–9^. Coin cells were assembled in open air by using graphite (15 mm) with the working electrode at the anode and 3 g of compost soil. Graphite was used as the counter electrode (cathode). Studies were carried out with five different concentrations of urea: 0.1 g/ml, 0.2 g/ml, 0.3 g/ml, 0.4 g/ml, and 0.5 g/ml. For comparison of power, the concentration of urea fuel was fixed at 0.5 g/ml in the liquid state. The dimension of the coin cell used for the experiment was that of CR2032 (30 d × 3.2 mm), and the surface area of the cell was 3.14 cm^2^. MPG-2 (Bio-Logic Science Instruments, France), with a 16-channel system, was used for coin cell study. The IV measurement of our soil-based fuel cell was done by Keithley 2420 source meter unit. The Keithley source meter generates the IV curve and provides vital parameters of a sample such as V_oc_ (open circuit voltage), I_sc_(short circuit current), I_max_, V_max_, P_max_. Figure 5 shows the curve of voltage vs current density and power density, which is, calculated from the IV data of SMU. The power density was later calculated by multiplying voltage and current density.

Even though the urea fuel cell was performed and here to optimise the role of the compost for the Bactria and enzymes, to understand the depth of the mechanistic details, additional bacteria colony counts studies done by using standard nutrient broth to see the growth of the microbes on samples with urea fuel treated samples 0.5 g/ml in the soil compost, and same another standard sample treated autoclaved sterilization study done at 120 °C, (killing the bacteria in the compost soil). This method provides complete information about forming the well-defined colonies. We took the 0.1 g soil sample from the coin cell. The experiment was used to demonstrate, and cultivability of the soil bacterium 0.1 g of soil (with distilled water, urea fuel 0.5 g) was first seeded into the 9 ml peptone saline diluent (PSD) for 1 hrs and incubated at fixed ambient temperature. Then inoculated PSD was further diluted into fresh PSD 1:9. The diluted soil suspension (100 µL) was directly distributed on the surface of the solid nutrient broth (NB) agar plates. After a few hours (0 to 28 hours), the growth of bacteria was checked after incubation at 37 °C.

### Electrochemical characterisation

Electrochemical properties of the compost soil were analysed by cyclic voltammeter (CV) studies performed in a bipolar mode, temperature-dependent CV studies, chronoamperometry study, cyclic stability studies, and electrochemical impedance spectroscopy (EIS) of the coin cell having compost soil study. The electrochemical performance of the SS compost soil with urea oxidation for the biobattery was optimized, and its function was studied using the MPG-2, 16 channel battery system cycle, (Bio-Logic Scientific Instrument, France).

For the electrical characterization, we chose a Keithley high current (SMU- Model-2420) interfaced with RS-232 mode, to study the different IV parameters. All the CV measurements were performed with graphite/graphite (Gr/Gr) electrodes in the same coin cell assembly.

## Supplementary information

Supplementary information.
