# Supplementary material for: Compost Soil Microbial Fuel Cell to Generate Power using Urea as Fuel
Source: Sci Rep. 2020 Mar 5;10:4154. doi: 10.1038/s41598-020-61038-7 (PMC7058052; doi:10.1038/s41598-020-61038-7)
Supplement: Supplementary file 1 — Supplementary information. [file 41598_2020_61038_MOESM1_ESM.docx]

***Supplementary information***

**Compost-based Solid State Microbial Fuel Cell to Generate Power using Urea as Fuel**

Verjesh Kumar Magotra^1^, Sunil Kumar^1^, T. W. Kang^1^, Akbar I. Inamdar^2^, Abu Talha Aqueel^2^, Hyunsik Im^2^, Gajanan Ghodake^3^, Surendra Krushna Shinde^3^, D. P. Waghmode^4^, H.C. Jeon^1^*

^1^Nano Information Technology Academy, Dongguk University-Seoul, Jung-Gu-100715, Seoul, South Korea

^2^Division of Physics and Semiconductor Science, Dongguk University, Jung-Gu-100715, Seoul, South Korea

^3^Department Biological and Environmental Science, College of Life Science and Biotechnology, Dongguk University-Seoul, Ilsandong-gu, 10326, Goyang-si, Gyeonggi-do, Republic of Korea

^4^Analytical Chemistry and Material Science Research Laboratory, Department of Chemistry, Shivaji University, Kolhapur 416004, Maharashtra, India

* hcjeon@dongguk.edu

**
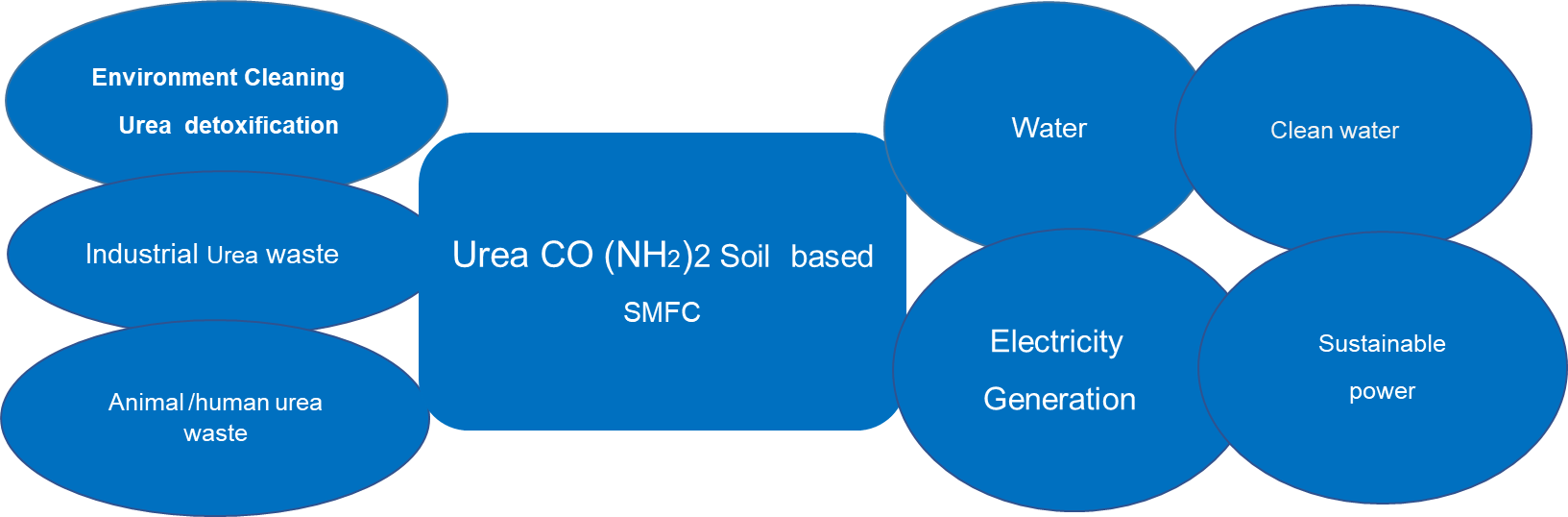
**

**Fig. S1**. The schematic diagram showing the vision of the paper for compost soil SMFC.


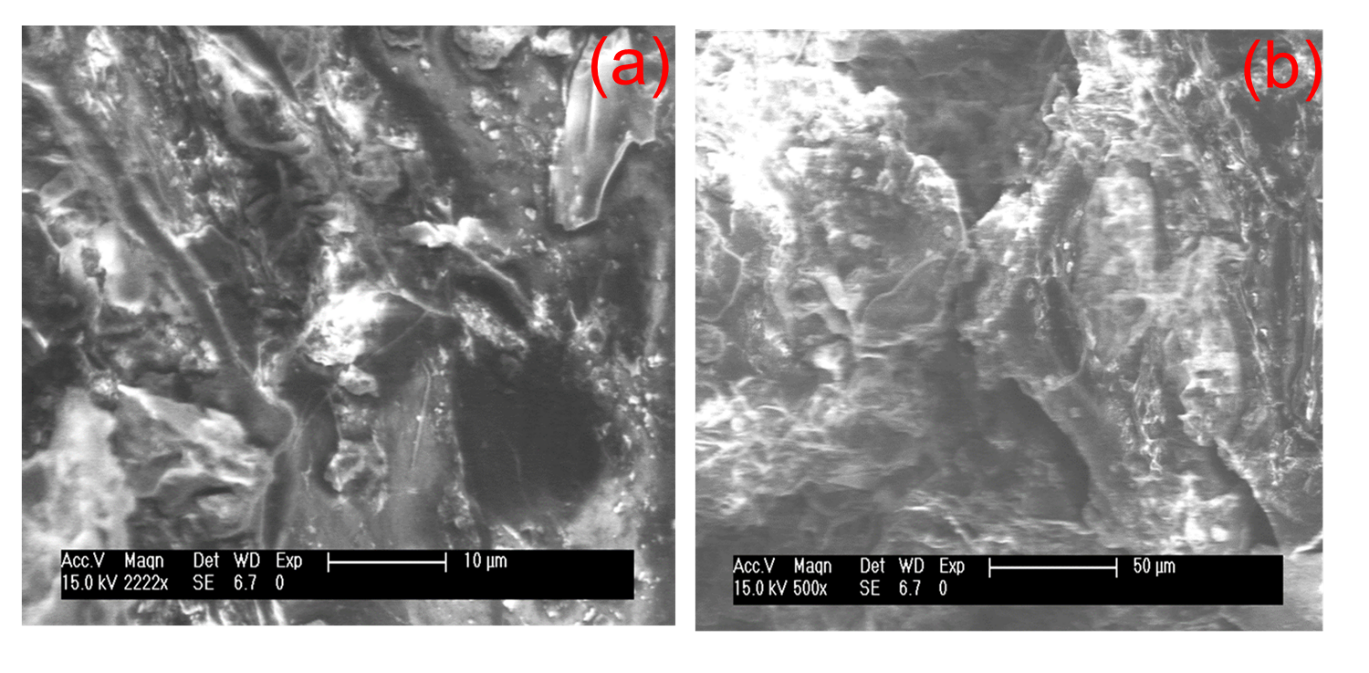


**Fig. S2 (a&b)** explain the SEM image to study the surface texture of the type of compost soil at different measurements 10 micrometres to 50 micrometres.

**
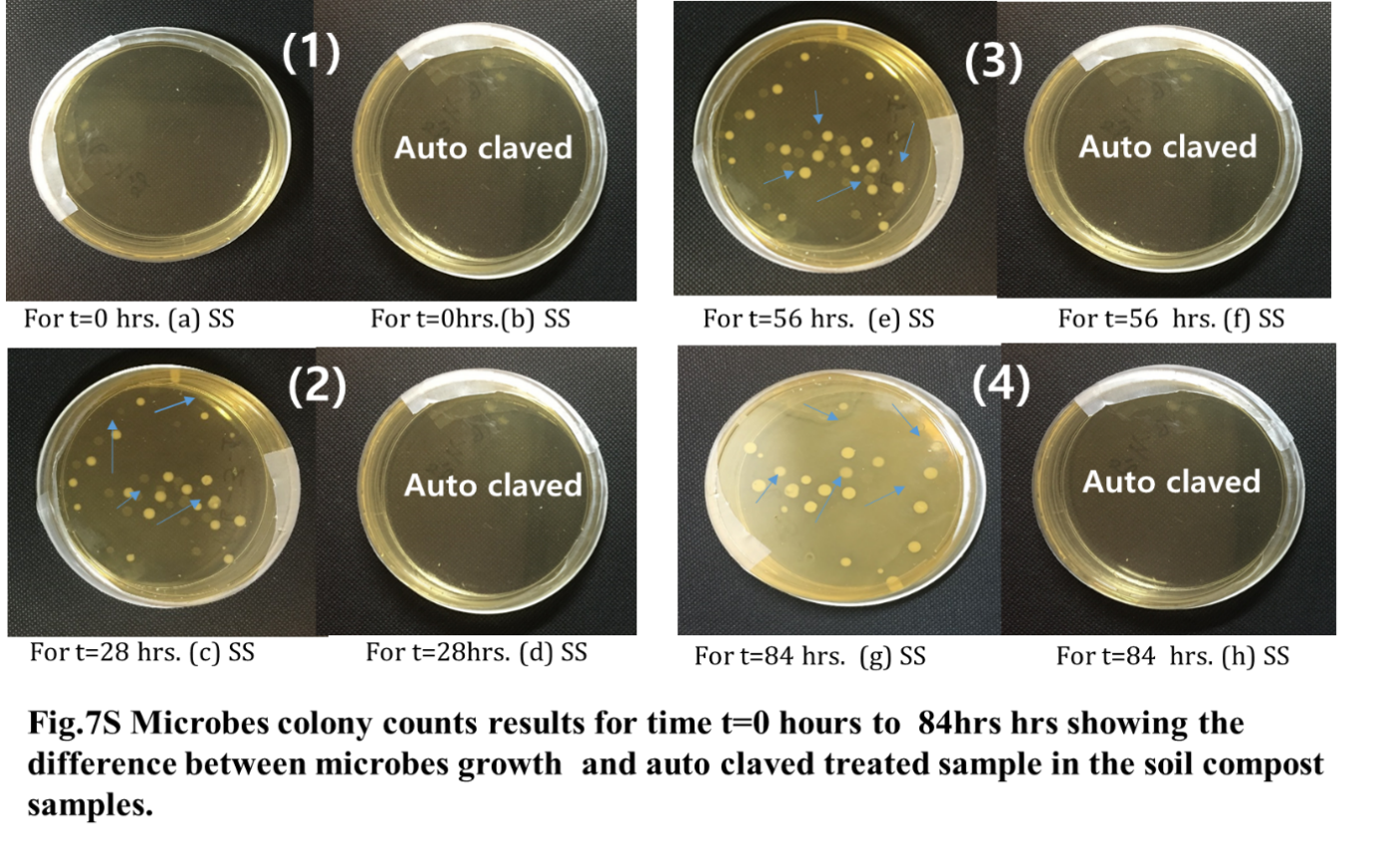
Fig.S3** Microbes colony counts results for time t=0 hours to 84hrs hrs showing the difference between microbes growth and autoclaved treated sample in the soil compost samples(SS).
